# Performance analysis of an adaptive optics system for free-space optics communication through atmospheric turbulence

**DOI:** 10.1038/s41598-018-19559-9

**Published:** 2018-01-18

**Authors:** Yukun Wang, Huanyu Xu, Dayu Li, Rui Wang, Chengbin Jin, Xianghui Yin, Shijie Gao, Quanquan Mu, Li Xuan, Zhaoliang Cao

**Affiliations:** 1 State Key Lab of Applied Optics, Changhun Institute of Applied Optics, Fine Mechanics and Physics, Chinese Academy of Sciences, Changchun, Jilin, China; 2 Chinese Academy of Sciences University, Beijing, China; 3 Changchun Institute of Optics, Fine Mechanics and Physics, Chinese Academy of Sciences, Changchun, Jilin, China

## Abstract

The performance of free-space optics communication (FSOC) is greatly degraded by atmospheric turbulence. Adaptive optics (AO) is an effective method for attenuating the influence. In this paper, the influence of the spatial and temporal characteristics of turbulence on the performance of AO in a FSOC system is investigated. Based on the Greenwood frequency (GF) and the ratio of receiver aperture diameter to atmospheric coherent length (*D*/*r*_*0*_), the relationship between FSOC performance (CE) and AO parameters (corrected Zernike modes number and bandwidth) is derived for the first time. Then, simulations and experiments are conducted to analyze the influence of AO parameters on FSOC performance under different GF and *D*/*r*_*0*_. The simulation and experimental results show that, for common turbulence conditions, the number of corrected Zernike modes can be fixed at 35 and the bandwidth of the AO system should be larger than the GF. Measurements of the bit error rate (BER) for moderate turbulence conditions (*D*/*r*_*0*_ = 10, *f*_*G*_ = 60 Hz) show that when the bandwidth is two times that of GF, the average BER is decreased by two orders of magnitude compared with *f*_*G*_/*f*_*3dB*_ = 1. These results and conclusions can provide important guidance in the design of an AO system for FSOC.

## Introduction

Free space optics communication (FSOC) can offer an alternative to Radio Frequency communication in modern wireless communication for its high data rate, high capacity, free license spectrum and excellent security^1^. However, the development of FSOC is limited by atmospheric turbulence. The amplitude fluctuation and wave-front distortion caused by atmospheric turbulence are the main factors that can severely degrade the coupling efficiency (CE) and increase the bit-error-rate (BER)^2^. To improve the performance of FSOC, an aperture averaging technique is typically used to attenuate the amplitude fluctuation^3^, with adaptive optics (AO) utilized to compensate for the wave-front phase distortion caused by atmospheric turbulence^4^. The traditional AO system is widely applied in the field of astronomical observation, which usually works at night in conditions of weak turbulence. A FSOC system must be able to maintain real-time data transmission even during periods of strong turbulence, such as sunrise, sundown, afternoon, etc. The data transmission rate in a FSOC system can reach Giga bit per second and above a 1 millisecond interruption will generate millions of bit errors. Such performance is intolerable in a realistic communication system. Hence, use of AO in a FSOC system demands higher correcting capability and system stability compared with AO used for astronomical observation.

In recent years, rapid progress has been made in studies of FSOC with AO. Many studies have focused on the feasibility of AO in the FSOC system and analyzed the performance of AO-based FSOC under different spatial or temporal atmospheric turbulence conditions. Tyson verified the effectiveness of AO for FSOC in theory and experiment. The use of AO can lead to a decrease in the BER of at least two orders of magnitude^5,6^. Takenaka and Toyoshima^7^ developed a simulation model of CE under atmospheric turbulence and verified the tracking performance of a fast steering mirror for ground-to-satellite links; the CE was improved by reducing the angle of arrival fluctuation. Huang Jian investigated the pattern of aberration modes for improving the BER of FSOC^8^. Chen Mo *et al*. studied the influence of atmospheric turbulence on CE over various turbulences conditions, and demonstrated an increase in the single mode fiber (SMF) CE from 1.3% to 46.1% after AO correction under strong turbulence^9^. Liu Chao *et al*. showed that AO is a powerful method for promoting BER performance by the compensation of spatial phase error,and indicated that the temporal phase error caused by the time delay of the AO correction loop also needs to be considered in FSOC^10^. Li *et al*.^11^ analyzed the effect of the temporal phase error using different Greenwood frequencies (GF); the servo bandwidth of the AO system in FSOC was also discussed. However, experimental analysis was ignored. Liu *et al*.^12^ experimentally studied the influence of the GF on FSOC performance, and the experimental results showed that a higher bandwidth is necessary to guarantee communication quality with increasing GF. Cao *et al*.^13^ analyzed FSOC performance based on experimental data for different GF and the ratio of receiver aperture diameter to atmospheric coherent length (*D*/*r*_*0*_); their results show that the influence of the atmospheric temporal characteristics on the FSOC performance is slightly stronger than that of the spatial characteristics. In practice, there are few analyses considering both the spatial characteristics (*D*/*r*_*0*_) and the temporal characteristics (GF); the quantitative relationship between the residual wave-front aberration of the AO and communication performance for FSOC is currently not clear. Therefore, the theoretical and experimental analysis for optimizing AO parameters is urgently required to be studied to improve the performance of FSOC under different spatial and temporal characteristics of atmospheric turbulence.

In this paper, the spatial and temporal characteristics of the residual wave-front aberration following AO correction are analyzed to evaluate the FSOC performance. First, the relationship between wave-front aberration and average CE is derived. Second, the relationship between AO parameters (number of corrected Zernike modes, bandwidth) and wave-front aberration after AO correction is derived under different conditions of atmospheric turbulence (GF, *D*/*r*_*0*_); then, the quantitative relationship between AO parameters and the communication performance of FSOC (BER, CE) is analyzed under different *D*/*r*_*0*_ and GF. Finally, an AO experimental platform is setup for the investigation of a FSOC system with a tip-tilt mirror (TTM) and a 145-element deformable mirror (DM). The CE and BER of this FSOC system with intensity modulation/direct detection (IM/DD) are calculated to evaluate the performance of the AO system for suppressing atmospheric turbulence in both spatial and temporal aspects. Lower bounds for the AO parameters for FSOC are obtained,which provide important guidance in AO system design for FSOC.

## Results

### Simulation Results

**Figure 1:**
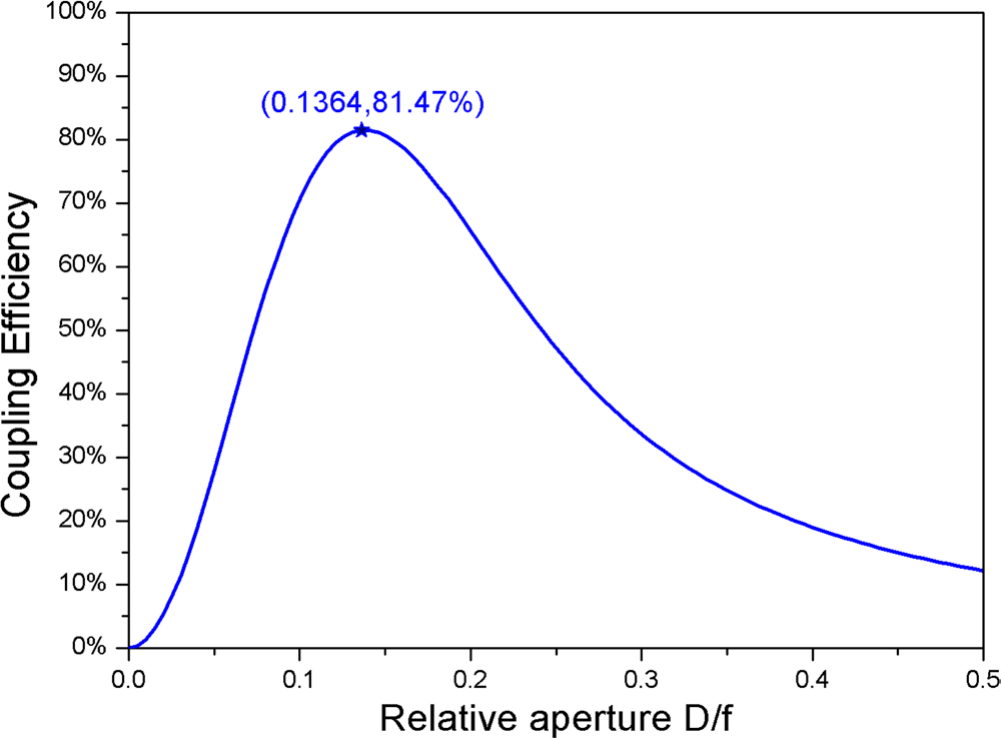
Relationship between CE and the relative aperture of the coupling lens.

In our FSOC, we use Pulse Position Modulation (PPM) with a bit-rate of 4 Gbps, receiver operation based on the direct-detection scheme, and uniform intensity in the detection plane. For a coupling system without atmospheric turbulence, the CE depends on the relative aperture of the coupling lens. The curve of CE versus relative aperture *D*/*f* of the coupling lens calculated using Eq. (8) is shown in Fig. 1. Given the radius of the optical fiber mode field w0 is 8 um, wavelength of the signal beam is 1550 nm, and receiving aperture is 12 mm, the maximum CE is obtained at an optimized *D*/*f* of 0.14. The BER can be calculated by Eq. (25); a BER below 10^−7^ is required to obtain an acceptable communication performance. Based on the theoretical analysis, the performance of AO for FSOC through atmospheric turbulence is studied by simulation of both spatial and temporal characteristics.

### Influence of the spatial characteristic of atmospheric turbulence

**Figure 2:**
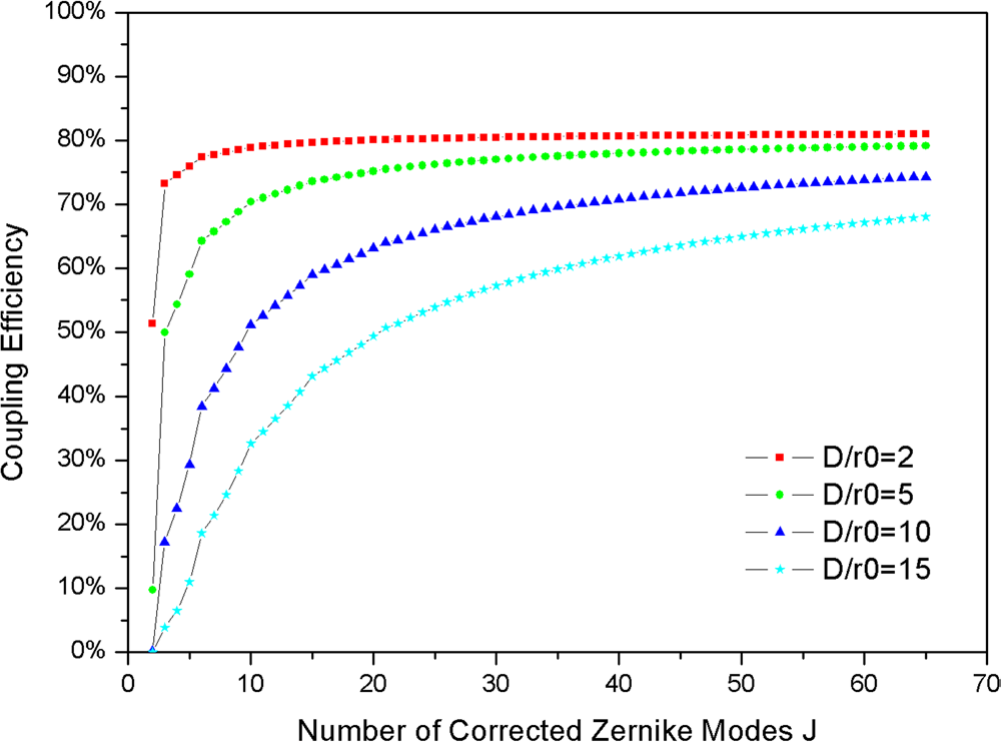
The relation between CE and number of corrected Zernike modes J under different *D*/*r*_*0*_.

For the AO system, we use the parameter-normalized atmospheric turbulence strength (*D*/*r*_*0*_) to represent the atmospheric turbulence spatial characteristic. For the weak turbulence condition, *D*/*r*_*0*_ is approximately 2. For the moderate turbulence condition, *D*/*r*_*0*_ is approximately 10. For the strong turbulence condition, *D*/*r*_*0*_ is approximately 15. From Eq. (19), the mean CE is influenced by the spatial characteristic *D*/*r*_*0*_ and temporal characteristic GF. To investigate the effect of AO on the spatial characteristic, we consider that the GF is zero to avoid the influence of the temporal characteristic. The relation between CE and the number of corrected Zernike modes J under different *D*/*r*_*0*_ is shown in Fig. 2.

As is shown in Fig. 2, CE increased with the number of corrected Zernike Modes for varying *D*/*r*_*0*_. For example, for weak atmospheric turbulence, *D*/*r*_*0*_ = 2, and the CE is higher than 50% with only tip-tilt correction, and increases to 80% with 15 Zernike modes corrected. Considering a stronger turbulence, *D*/*r*_*0*_ = 5, and the CE is 50.1% with 3 Zernike modes corrected. Therefore, under the condition of weak turbulence, *D*/*r*_*0*_ < 5, and the CE is higher than 50% with only low order Zernike modes corrected. However, for moderate or strong turbulence, 30 or more Zernike modes should be corrected to improve the CE from nearly zero to 50%. In particular, for strong turbulence, *D*/*r*_*0*_ = 15, the CE can only reach a maximum of 68% with 65 Zernike Modes corrected.

For a FSOC system, the final evaluation of the communication performance is the BER. The BER is influenced by the light power at the receiving aperture, sensitivity of the detector and the CE. To realize an acceptable level of communication performance (BER < 10^−7^), the lower bound for the CE is different in different FSOC systems. With the requirement of minimum CE in different FSOC system, we can obtain the minimum number of corrected Zernike modes under different atmospheric turbulence strengths. In the AO system, the sub-aperture number of WFS determines the number of Zernike modes that can be measured, and the element number of DM determines the number of Zernike modes that can be corrected. Hence, the data shown in Fig. 2 can be used to assist selection of the sub-aperture number for WFS and the element number for DM. Generally, the number of corrected Zernike modes fixed at 35 is sufficient for common turbulence conditions (*D*/*r*_*0*_ < 15).

Therefore, the influence of the spatial characteristic of atmospheric turbulence can be analyzed as described above. In addition to the spatial characteristic, the temporal characteristic of the atmospheric turbulence is another crucial factor that can strongly affect the FSOC performance.

### Influence of the temporal characteristic of atmospheric turbulence

**Figure 3:**
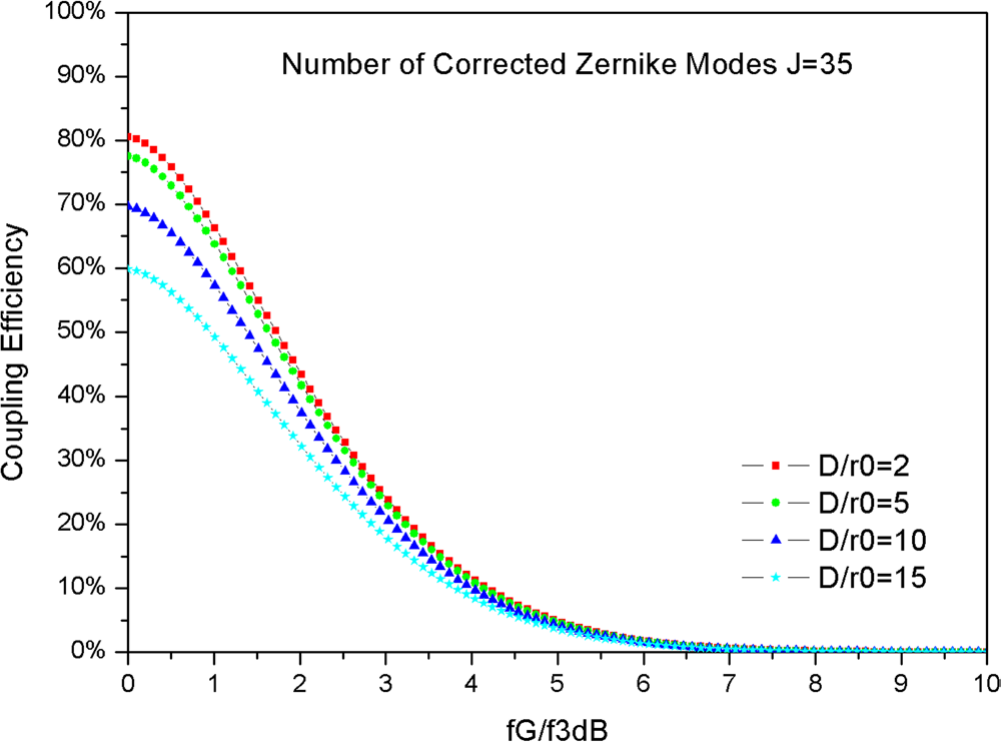
Curve of CE versus *f*_*G*_*/f*_*3dB*_ for varying *D*/*r*_*0*_.

The GF (*f*_*G*_) is defined to represent the temporal characteristic of atmospheric turbulence, and the −3 *dB* closed loop bandwidth of AO (*f*_*3dB*_) is used to describe the temporal characteristic of the AO system. Hence, the ratio *f*_*G*_/*f*_*3dB*_ is used to represent the temporal characteristic of wave-front aberrations following AO correction. Figure 3 shows the curve of CE versus *f*_*G*_*/f*_*3dB*_ calculated using Eq. (19) for varying *D*/*r*_*0*_ and the number of corrected Zernike modes J is 35.

As shown by the data in Fig. 3, the CE decreases with increasing *f*_*G*_/*f*_*3dB*_ for different *D*/*r*_*0*_. When the *f*_*G*_*/f*_*3dB*_ is less than 1, the CE is higher than 50% under different *D*/*r*_*0*_. However, when *f*_*G*_*/f*_*3dB*_ is larger than 1, the CE decreases rapidly. In particular, when *f*_*G*_*/f*_*3dB*_ is five, the CE is only 3% for all *D*/*r*_*0*_ conditions. Generally, the maximum number of corrected Zernike modes for the AO system is fixed. When the number of corrected Zernike modes is 35, the *f*_*G*_*/f*_*3dB*_ should be designed to meet the performance requirement under different *D*/*r*_*0*_ to reach the minimum value of the CE. For a minimum CE of 30%, the GF may be similar to or larger than the bandwidth of AO under any *D*/*r*_*0*_. However, for a minimum CE of 50%, the GF cannot be larger than 1.8 times the bandwidth of AO under weak turbulence (*D*/*r*_*0*_ = 2). Considering strong turbulence (*D*/*r*_*0*_ = 15), the GF cannot be larger than 1.2 times the bandwidth of AO.

In the AO system, the bandwidth is limited by the sampling rate of the WFS, the resonant frequency of the wave-front corrector and the control parameters. The data shown in Fig. 3 are useful for the bandwidth design of an AO system. Therefore, the bandwidth of the AO should be larger than the GF to realize a good coupling performance and should not be less than half of the GF to obtain an available coupling performance.

In our simulation, the quantum efficiency is 0.85, mean power of the detector noise is 0.001, and the load resistance of the detector is 3.5 K ohms; form Eq. (25), the CE must be above 50% to obtain an acceptable BER performance (10^−7^) in our simulation. With the number of corrected Zernike modes is 35, system performance is sufficient to afford common atmospheric turbulence (*D*/*r*_*0*_ < 15); the minimum bandwidth of AO is 1.04 times that of GF for *D*/*r*_*0*_ = 15, and is 0.58 times that of GF for *D*/*r*_*0*_ = 2. Therefore, lower bounds for the AO system parameters for FSOC are obtained under both spatial and temporal characteristic turbulence conditions. The results from our simulations are very important for the design of an AO system for FSOC.

### Experiment Results

**Figure 4:**
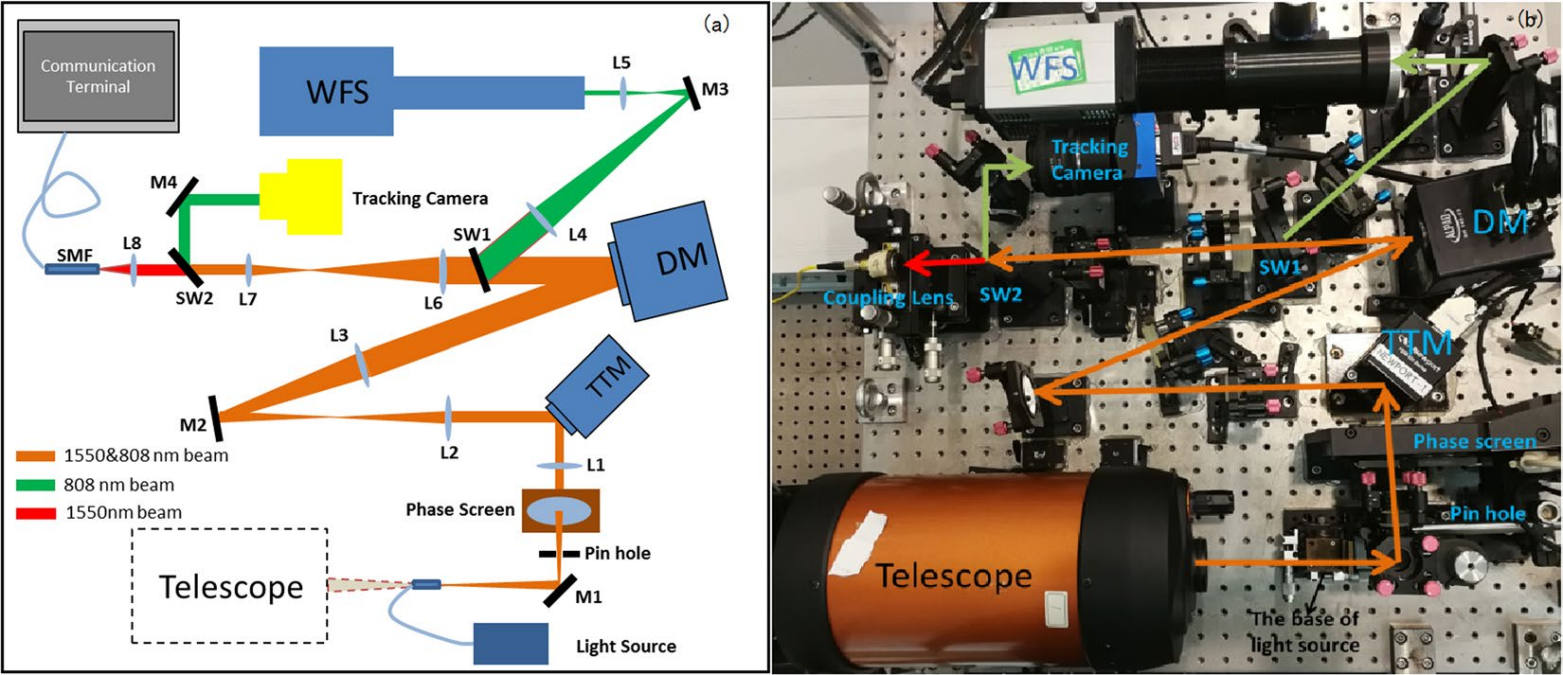
The experimental optical layout of the AO system for FSOC: (**a**) schematic diagram; (**b**) photograph.

To further investigate the performance of AO for FSOC through atmospheric turbulence, and verify the simulation results, an experimental FSOC system with AO is setup using a tilt-tilt mirror (TTM, from Newport Corporation), 145-element deformable mirror (DM, from ALPAO Corporation), and a Hartman wave-front sensor (WFS, built in-house) with a frame rate of 1.6 kHz. The WFS operates at 808 nm, with the residual wave-front aberration data transformed at 1550 nm to be consistent with the communication wavelength. A schematic diagram and photograph of the experimental system are shown in Fig. 4.

As shown in Fig. 4, the telescope is designed for receiving a light beam from the transmitter, but is replaced by a light source in this experiment. The light source consists of an 808 nm beacon beam and 1550 nm signal beam. M1, M2 M3 and M4 are reflective mirrors. The transmitted beam is collimated by the lens L1 with a phase screen placed into the path of the collimated beam. The TTM and the tracking camera are conjugated to measure and correct for the tip/tilt modes in the wave-front aberration. Then, the beam passes through the DM and is split into two parts by SW1. The 808 nm beam is 80% reflected by SW1, before finally entering the WFS. The WFS and the DM are conjugated. The transmitted beam passing through SW1 arrives at SW2, with the remaining component of the 808 nm beam 100% reflected by SW2. Then, the 1550 nm beam passes through SW2 and enters the coupling lens, which couples the light into the fiber. To simulate different atmospheric turbulence conditions, a phase screen from Lexitek Corporation is used that satisfies the Kolmogorov turbulence theory. We can change *r*_*0*_ through adjusting the aperture size of the pin hole near the phase screen:

1 $$\frac{D}{{r}_{0}}=\frac{{D}_{p}}{{r}_{0p}}$$

where *D* is the receiving antenna aperture, ${r}_{0p}$ is the coherent length of the phase screen, and *D*_*p*_ is the effective aperture size of the phase screen. In our experimental system, ${r}_{0p}$ is 1.08 mm. For example, when *D*_*p*_ is 10 mm, the spatial characteristic of the atmospheric turbulence (*D*/*r*_*0*_) is 9.2. In addition, the GF can be changed by changing the phase screen rotation speed.

**Figure 5:**
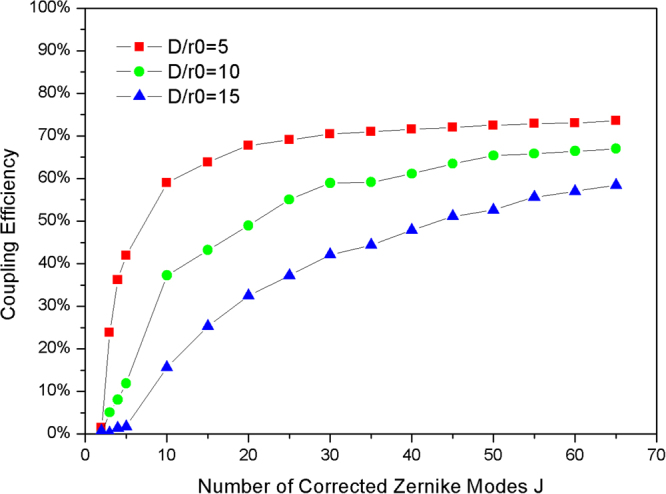
The experimental relationship between CE and number of corrected Zernike modes.

To investigate the influence of the spatial characteristic of atmospheric turbulence and obtain the relationship between CE and number of Zernike modes corrected by AO, the speed of the phase screen rotation is set to zero, and *D*/*r*_*0*_ is adjusted via the pin hole. In particular, the effective aperture size of the phase screen is fixed by the pin hole; next, the wave-front aberrations are corrected by the AO system; the number of corrected Zernike modes is set from 2 to 65 in the AO system; after AO correction, the residual wave-front aberrations are measured for the calculation of the CE. Then, the effective aperture size of the phase screen is changed to simulate different conditions of atmospheric turbulence, and the above-described AO system adjustments repeated. The experimental results are shown in Fig. 5.

**Figure 6:**
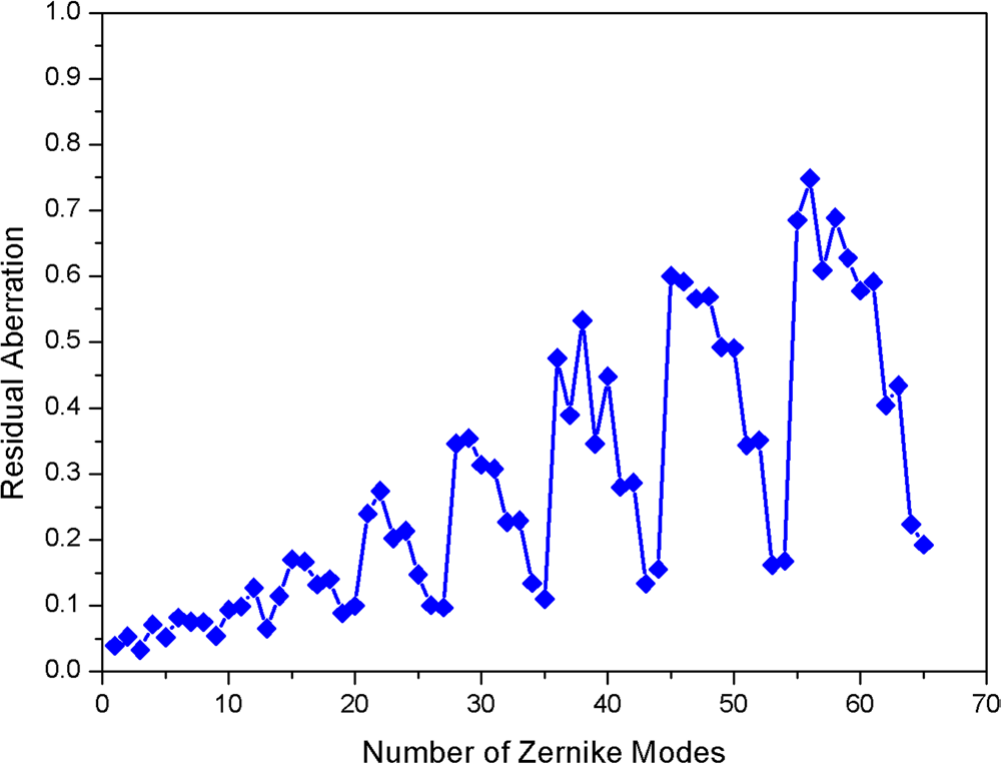
The residual aberration versus number of Zernike modes after correction by the AO system.

Figure 5 shows the average CE after AO correction as measured for different *D*/*r*_*0*_. The CE is improved with increasing number of corrected Zernike modes. The experimental CE is lower than the theoretical value indicated in Fig. 2. This difference is due to the transmittance limitation of the lens, wave-front measuring errors, and DM fitting errors. As shown in Fig. 6, the residual aberration due to the Zernike modes is not fully corrected by the AO system, and it is fluctuant with different Zernike Modes. Hence, the difference between the experimental results and the theoretical results is acceptable.

**Figure 7:**
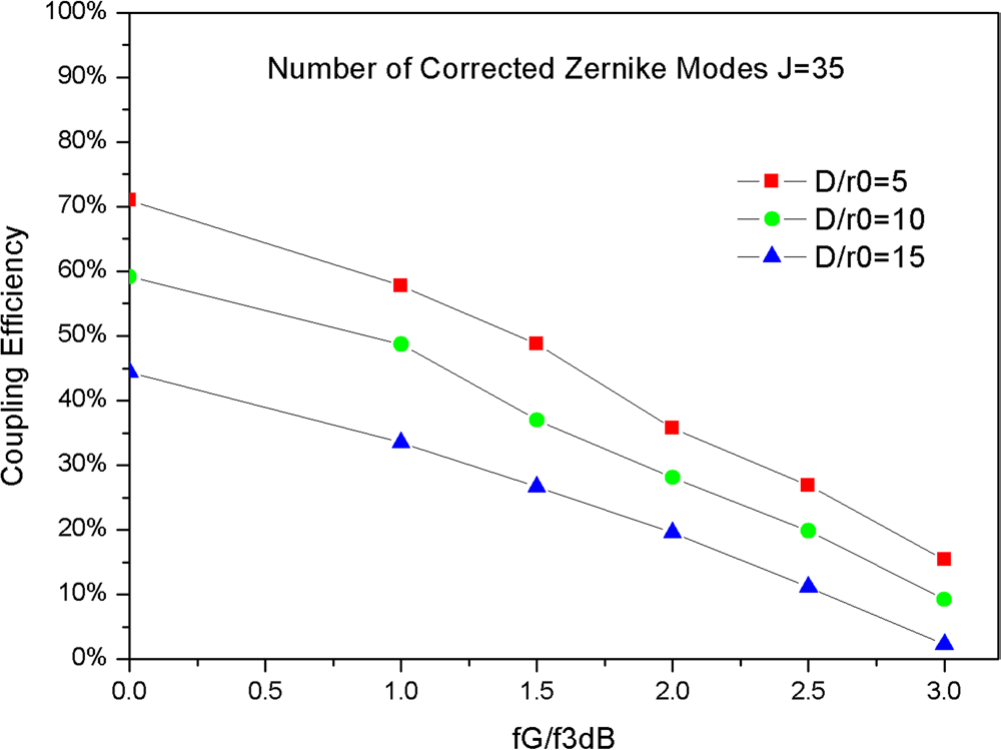
The experimental relationship between *f*_*G*_*/f*_*3dB*_ and CE under different *D*/*r*_*0*_.

Next, the influence of the temporal characteristic of atmospheric turbulence is experimentally studied. The GF is adjusted by changing the rotation speed of the phase screen; the maximum GF that can be generated is 180 Hz. Hence, the measurement range for the GF is from 0 Hz to 180 Hz in our experiment. The bandwidth of the AO system is set to 60 Hz, the number of corrected Zernike modes is set to 35, with the residual wave-front aberrations following AO correction measured for varying GF. The experimental results including the measured CE are shown in Fig. 7.

**Figure 8:**
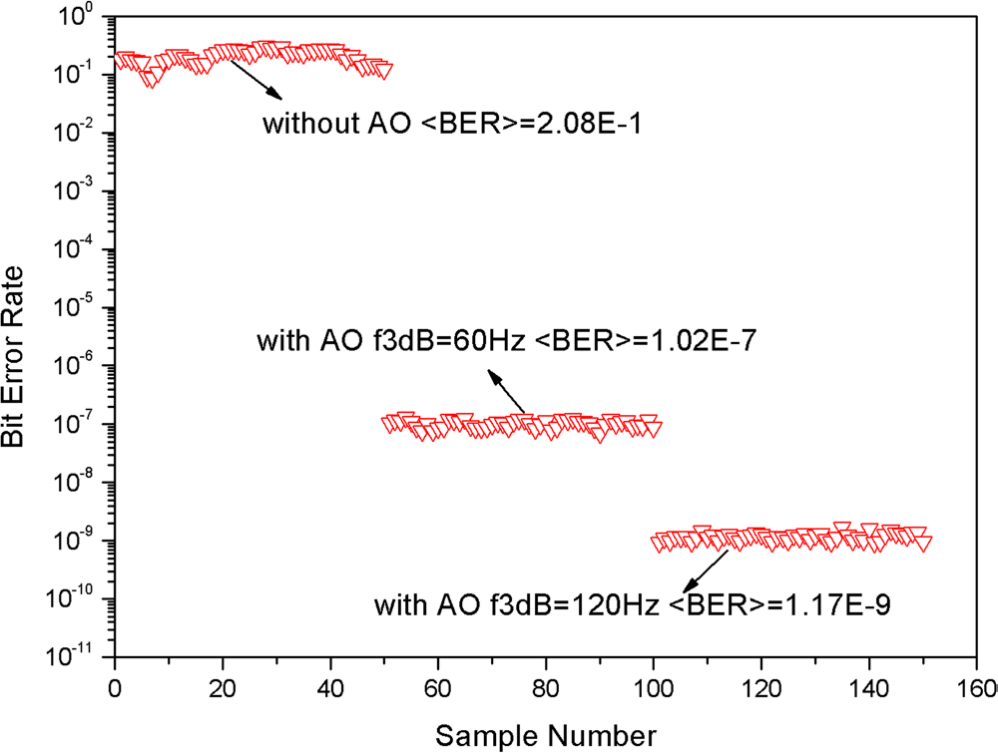
BER obtained under moderate turbulence conditions (*D*/*r*_*0*_ = 10, *f*_*G*_ = 60 Hz).

The experimental data indicate the maximum value of *f*_*G*_*/f*_*3dB*_ obtained under different *D*/*r*_*0*_, as shown in Fig. 7. For the condition of strong atmospheric turbulence (*D*/*r*_*0*_ = 15), the max CE is less than 50%, which indicates that a larger number of Zernike modes should be corrected to meet an acceptable level of communication performance (CE > 50%) for our FSOC. The BER was also measured, with and without AO correction, under a moderate turbulence condition (*D*/*r*_*0*_ = 10, *f*_*G*_ = 60 Hz); the results are shown in Fig. 8. The average BER is 2.08E-1 without AO correction, which indicates that the communication is interruptive. When the bandwidth of the AO system equals GF, the average BER with AO correction becomes 1.02E-7; however, when the bandwidth of the AO system is two times that of GF, the average BER is 1.17E-9, i.e., decreasing by approximately two orders of magnitude compared with *f*_*G*_*/f*_*3dB*_ = 1.

Therefore, the AO system for FSOC is effective in improving the communication performance. Only low orders of Zernike modes should be corrected for weak atmospheric turbulence; however, for increasing strength of atmospheric turbulence, more Zernike modes should be corrected. The analysis of the GF and bandwidth indicates a lower bound for the AO system bandwidth. A higher bandwidth for the AO system favors better communication performance. The experiments verified the theoretical simulation well. Although the experimental results are inferior to the simulation results due to spatial fitting errors and temporal dynamic errors, the conclusions from the simulation and experimental studies are consistent with each other. The simulation and experiment results both show lower bounds for the AO parameters under different GF and *D*/*r*_*0*_. The results of this paper are significant in the design of an AO system for FSOC.

## Discussion

In this paper, the influence of spatial and temporal characteristics of atmospheric turbulence for FSOC is investigated. Based on theoretical analysis of FSOC coupling system and AO system, the quantitative relationship between AO parameters (number of corrected Zernike modes and bandwidth) and communication performance (CE and BER) is derived for the first time. Then, simulations and experiments are carried out to analyze the influence of different spatial and temporal atmospheric conditions. The simulation results and experimental results give lower bounds for the AO parameters, and demonstrate the relationship between FSOC performance, bandwidth, *D*/*r*_*0*_, and GF. The lower bounds for AO parameters can be used to guarantee the quality of communication. For common turbulence conditions (*D*/*r*_*0*_ < 15), the number of corrected Zernike modes can be fixed at 35; the bandwidth of the AO should be larger than the GF to realize a good coupling performance and should not be less than half of the GF to obtain an available coupling performance. The influence of the atmospheric temporal characteristics on FSOC performance is slightly stronger than that of the spatial characteristics, a higher bandwidth is necessary to guarantee the quality of communication with increasing GF.

For further work, a field experiment will be conducted and the system performance will be further improved by increasing the bandwidth through advanced control methods. The conclusions of this paper can offer theoretical and experimental guidance in the design of an AO system for FSOC.

## Methods

**Figure 9:**
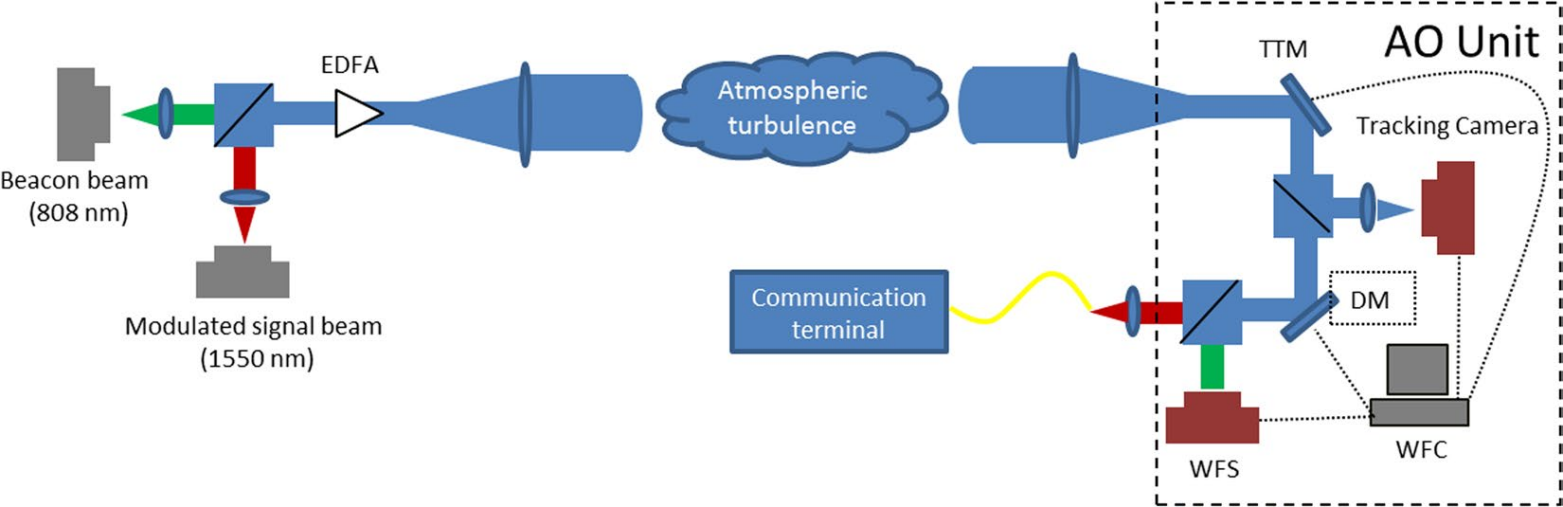
Schematic diagram of FSOC with AO system.

In this section, we briefly describe the derivation of the relationship between AO parameters and communication performance. A schematic diagram of FSOC with AO used to compensate for atmospheric turbulence is shown in Fig. 9. The system consists of a transmitter, receiver with AO, and atmospheric turbulence simulator. The signal beam is modulated and simultaneously joined with the beacon beam by a beam splitter before amplification by an erbium doped fiber amplifier (EDFA) and emission through the transmitter antenna. Then, the two beams are transmitted through the atmospheric turbulence simulator before arriving at the receiving terminal. After beam alignment, the beams pass through the AO system and any wave-front aberrations caused by atmospheric turbulence are compensated. The beam is split into a beacon beam and signal beam via the beam splitter. The beacon beam enters the wave-front sensor for measurement of wave-front aberrations. The wave-front controller controls the wave-front corrector according to the measured wave-front aberrations. Next, the distorted wave-front is corrected by the wave-front corrector. The signal beam is coupled into a SMF and demodulated by the communication terminal. The CE of FSOC through atmospheric turbulence and the performance of AO in FSOC are discussed as follows.

### The CE of FSOC through atmospheric turbulence

**Figure 10:**
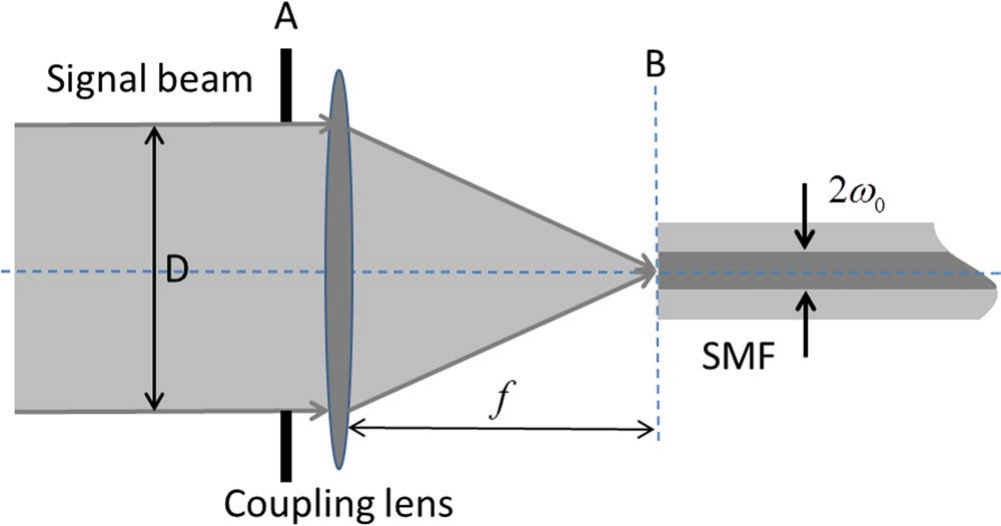
Geometrical diagram of optical coupling.

The CE is an important parameter in a FSOC system. It determines the light power that can be detected. The geometrical diagram of an optical coupling system is shown in Fig. 10. After application of the aperture averaging technique, the intensity of the signal beam received can be considered as uniform, and the wave-front phase is distorted by atmospheric turbulence, the signal beam is focused by a coupling lens with an effective aperture diameter of *D* and a focal length *f*. The lens is located in the plane A and the fiber is located in the focus plane B of the lens. The optical field distributions of the signal beam at plane A can be expressed as^14^

2 $${E}_{A}(x,y)={E}_{s}(x,y)\exp (-j\phi (x,y))$$

where *x*, *y* are the two-dimensional space coordinates, $\phi (x,y)$ is the wave-front phase induced by atmospheric turbulence, ${E}_{s}(x,y)$ is the aperture function, which, represents the intensity of the optical field.

The fundamental mode of power distribution in an SMF can be approximated within 1% error by a Gaussian beam if the normalized frequency V of the optical fiber is in the range 1.9 < V < 2.4. Based on this condition, the Gaussian approximation can be used to describe the optical fiber mode field distribution characteristics. The CE is used to describe the similarity between the optical field of the focused signal beam and the optical fiber mode field. If the CE at plane A is the same as at plane B, assuming the optical fiber mode field back-propagates to plane A to facilitate the CE calculation, the optical fiber mode field *F*_*A*_(*x*, *y*) at plane A is given by the expression^9^

3 $${F}_{A}(x,y)=\sqrt{\frac{2}{\pi {w}_{\alpha }^{2}}}\exp (-\frac{{x}^{2}+{y}^{2}}{{w}_{\alpha }^{2}})$$

where ${w}_{\alpha }=\frac{\lambda f}{\pi {w}_{0}}$ is the radius of the optical fiber mode field at plane A, *λ* is the wavelength, *f* is the lens focus length, and *w*_0_ is the radius of the optical fiber mode field at plane B. The CE *η* is defined as the ratio of the average power coupled into the fiber $\langle {P}_{f}\rangle$ to the average power received at the aperture plane $\langle {P}_{a}\rangle$, and is given by

4 $$\begin{array}{rcl}\eta =\frac{\langle {P}_{f}\rangle }{\langle {P}_{a}\rangle } & = & \frac{{|{\iint }_{S}{E}_{A}^{\ast }({\rm{x}},{\rm{y}}){{\rm{F}}}_{A}(x,y)ds|}^{2}}{{\iint }_{S}{|{E}_{A}(x,y)|}^{2}ds\cdot {\iint }_{S}{|{F}_{A}(x,y)|}^{2}ds}\\  & = & \frac{1}{S}{|{\iint }_{S}\exp [-j\phi (x,y)]\cdot \sqrt{\frac{2}{\pi {w}_{\alpha }^{2}}}\exp (-\frac{{x}^{2}+{y}^{2}}{{w}_{\alpha }^{2}})ds|}^{2}\\  & = & \frac{8}{{\pi }^{2}{D}^{2}{w}_{\alpha }^{2}}|{\iint }_{s}\exp (-\frac{{x}^{2}+{y}^{2}}{{w}_{\alpha }^{2}})\cos (\phi ({\rm{x}},{\rm{y}})){\rm{ds}}-{\rm{j}}\\  &  & {\iint }_{S}{\exp (-\frac{{x}^{2}+{y}^{2}}{{w}_{\alpha }^{2}})\sin (\phi ({\rm{x}},{\rm{y}})){\rm{ds}}|}^{2}\end{array}$$

The expression for CE can be rewritten as

5 $$\eta =\frac{8}{{\pi }^{2}{D}^{2}{w}_{\alpha }^{2}}{|a|}^{2}$$

where $a={\iint }_{S}\exp (-\frac{{x}^{2}+{y}^{2}}{{w}_{\alpha }^{2}})\cos (\phi ({\rm{x}},{\rm{y}})){\rm{ds}}-j{\iint }_{S}\exp (-\frac{{x}^{2}+{y}^{2}}{{w}_{\alpha }^{2}})\sin (\phi ({\rm{x}},{\rm{y}})){\rm{ds}}$, $S=\pi {D}^{2}/4$ is the effective area of the receiving aperture, and ${\iint }_{S}{|{F}_{o}(x,y)|}^{2}{\rm{ds}}=1$ satisfies the fiber normalization condition.

The value of *a* obeys the normal distribution, with a probability density functionthat can be expressed as

6 $$P(a)=\frac{1}{\sqrt{2\pi }\sigma }\exp [-\frac{{(a-\bar{a})}^{2}}{2{\sigma }^{2}}]$$

where *σ* is the variance of *a*, $\bar{a}$ is the mean value of *a*. After calculation, we can easily obtain the following expression for $\bar{a}$:

7 $$\bar{a}={\iint }_{S}\exp (-\frac{{x}^{2}+{y}^{2}}{{w}_{\alpha }^{2}})ds\,\exp (-\frac{1}{2}{\sigma }_{\phi }^{2})$$

where ${\sigma }_{\phi }$ is the root mean square (RMS) of $\phi (x,y)$. Hence, the average value of CE is

8 $$\bar{\eta }=\frac{8}{{\pi }^{2}{D}^{2}{w}_{\alpha }^{2}}{|{\iint }_{S}\exp (-\frac{{x}^{2}+{y}^{2}}{{w}_{\alpha }^{2}})ds\exp (-\frac{1}{2}{\sigma }_{\phi }^{2})|}^{2}$$

Thus, the relationship between wave-front aberrations and average CE is established together with the fiber coupling theory. Next, we derive the residual wave-front aberration after AO correction using the power spectrum for atmospheric turbulence and the AO correction model.

### The residual wave-front aberrations after AO correction

To analyze the influence of the CE in FSOC with AO correction, it is important to derive the RMS of the wave-front aberration based on the models for AO and atmospheric turbulence. The close loop transfer function of the AO system can be expressed as^15,16^

9 $$H(f)=\frac{1}{1+jf/{f}_{3dB}}$$

where ${f}_{3dB}$ is the closed loop bandwidth of the AO system.

After AO correction, the residual wave-front aberration is

10 $${\sigma }_{\tau }^{2}={\int }_{0}^{\infty }{|1-H(f)|}^{2}{P}_{psd}(f)df$$

where *f* is the cyclic frequency of the atmospheric turbulence. ${P}_{psd}(f)$ is the power spectrum of the atmospheric turbulence, which can be described as^16^

11 $${P}_{psd}(f)=0.0326{(\frac{2\pi }{\lambda })}^{2}{f}^{-8/3}{\int }_{0}^{L}{C}_{n}^{2}(z){V}^{5/3}(z)dz$$

where *L* is the path length, *λ* is the wavelength for communication, *V*(*z*) is the model for the wind speed, and ${C}_{n}^{2}(z)$ represents the atmospheric refractive index structure parameter.

To solve Eq. (10), we used a binary filter given by

12 $$H^{\prime} (f)=\{\begin{array}{cc}1 & f\le {f}_{eq}\\ 0 & f > {f}_{eq}\end{array}$$

where ${f}_{eq}$ is the equivalent close loop bandwidth of AO, which can be solved by

13 $${\int }_{0}^{{f}_{eq}}H^{\prime} (f)df={\int }_{0}^{{f}_{3dB}}H(f)df\Rightarrow {f}_{eq}={\int }_{0}^{{f}_{3dB}}H(f)df$$

Hence,

14 $${\sigma }_{\tau }^{2}={\int }_{{f}_{eq}}^{\infty }{P}_{psd}(f)df=\frac{0.0784{\pi }^{2}}{{\lambda }^{2}}{\{{\int }_{0}^{{f}_{3dB}}H(f)df\}}^{-5/3}{\int }_{0}^{L}{C}_{n}^{2}(z){V}^{5/3}(z)dz$$

The temporal characteristic of the atmospheric turbulence can be described by GF, which can be expressed by^16^

15 $${f}_{G}={[0.102{(\frac{2\pi }{\lambda })}^{2}{\int }_{0}^{L}{C}_{n}^{2}(z){V}^{5/3}(z)dz]}^{3/5}$$

After integrating Eqs (14) and (15), the expression for theresidual wave-front aberration can be rewritten as

16 $${\sigma }_{\tau }^{2}={(\frac{{f}_{G}}{{f}_{3dB}})}^{5/3}$$

In atmospheric turbulence theory, the wave-front is usually described by the Zernike polynomial, the AO system can only correct finite Zernike modes, with aspatial fitting error remaining. Assuming that the front *J* modes are corrected by AO, the residual spatial fitting error ${\sigma }_{J}^{2}$ can be described as^17^

17 $${\sigma }_{J}^{2}={C}_{J}{(\frac{D}{{r}_{0}})}^{5/3}$$

where *C*_*J*_ is a coefficient of Zernike fitting error, *C*_*J*_ is decreasing with the increasing of *J*.*D* is the receiving aperture, and *r*_*0*_ is the atmospheric coherent length. Hence, the total RMS of the residual wave-front aberration is given by

18 $${\sigma }_{\phi }^{2}={\sigma }_{J}^{2}+{\sigma }_{\tau }^{2}={C}_{J}{(\frac{D}{{r}_{0}})}^{5/3}+{(\frac{{f}_{G}}{{f}_{3dB}})}^{5/3},$$

Combing Eqs (8) and (18), the average CE is given by

19 $$\bar{\eta }=\frac{8}{{\pi }^{2}{D}^{2}{w}_{\alpha }^{2}}{|{\iint }_{S}\exp (-\frac{{x}^{2}+{y}^{2}}{{w}_{\alpha }^{2}})ds\exp (-\frac{1}{2}({C}_{J}{(\frac{D}{{r}_{0}})}^{5/3}+{(\frac{{f}_{G}}{{f}_{3dB}})}^{5/3}))|}^{2}$$

### The BER of a FSOC system

In the FSOC system, the BER is given by

20 $$BER=\frac{1}{2}erfc(\frac{\sqrt{SNR}}{2})$$

where $erfc(\bullet )$ is the complementary error function, and *SNR* is the signal to noise ratio for detection. The optical power at the receiver is given by

21 $$P={{\iint }_{S}|{E}_{A}(x,y)|}^{2}ds$$

The power coupled into the fiber is

22 $$P^{\prime} =P\cdot \bar{\eta }$$

The power detected by the detector is

23 $${S}_{P}={(\frac{e\delta }{hv})}^{2}{P^{\prime} }^{2}{R}_{L}$$

where *e* is the electron charge, *δ* is the quantum efficiency of the detector, *h* is Planck’s constant, *v* is the frequency of the light, and *R*_*L*_ is the load resistance of the detector. The SNR can be written as

24 $$SNR=\frac{{S}_{p}}{{\sigma }_{n}^{2}}$$

where ${\sigma }_{n}^{2}$ is the average power of the detector noise. Hence, the BER of the FSOC is given by

25 $$BER=\frac{1}{2}erfc(\frac{|\frac{e\delta }{hv}\frac{P\cdot \bar{\eta }}{{\sigma }_{n}}|\sqrt{{R}_{L}}}{2})$$

Thus, the relationship between AO parameters and FSOC performance is obtained, and the theoretical derivation is complete.

## References

[1] Majumdar, A. K., Ricklin, J. C. Free-space laser communications: principles and advances 5–10 (Springer, 2010).

[2] Andrews, L. C. & Phillips, R. L. Laser Beam Propagation through Random Media 9–15 (SPIE Press, 2005).

[3] Lee IE, Ghassemlooy Z, Ng WP, Khalighi MA, Liaw SK (2016). Effects of aperture averaging and beam width on a partially coherent Gaussian beam over free-space optical links with turbulence and pointing errors. Applied Optics.

[4] Leonhard N, Berlich R, Minardi S (2016). Real-time adaptive optics testbed to investigate point-ahead angle in pre-compensation of Earth-to-GEO optical communication. Optics Express.

[5] Tyson RK (2002). Bit-error rate for free-space adaptive optics laser communications. J. Opt. Soc. Am..

[6] Tyson RK, Canning DE, Tharp JS (2005). Measurement of the bit-error rate of an adaptive optics, free-space laser communications system, part1: tip-tilt configuration, diagnostics, and closed-loop results. Optical Engineering.

[7] Takenaka, H. & Toyoshima, M. Study on the fiber coupling efficiency for ground-to-satellite laser communication links. *Proc. Of Spie*, **7587U** (2010).

[8] Jian H (2014). Effectiveness of adaptive optics system in satellite-to-ground coherent optical communication. Optics Express.

[9] Chen M, Liu C, Xian H (2015). Experimental demonstration of single-mode fiber coupling over relative strong turbulence with adaptive optics. Applied Optics.

[10] Liu C, Chen S, Li XY, Xian H (2014). Performance evaluation of adaptive optics for atmospheric coherent laser communications. Applied Optics.

[11] Li J, Zhang Z, Gao J, Sun J, Chen W (2016). Bandwidth of adaptive optics system in atmospheric coherent laser communication. Optics Communications.

[12] Liu W (2016). Performance evaluation of coherent free space optical communications with a double-stage fast steering- mirror adaptive optics system depending on the Greenwood frequency. Optics Express.

[13] Cao J, Zhao X, Liu W, Gui H (2017). Performance analysis of a coherent free space optical communication system based on experiment. Optics Express.

[14] Takenaka H, Toyoshima M, Takayama Y (2012). Experimental verification of fiber-coupling efficiency for satellite-to-ground atmospheric laser downlinks. Optics Express.

[15] Wang Y (2017). High-precision identification of a tip–tilt control system for the compensation of time delay. Applied Optics.

[16] Darryl P (1977). Greenwood. Bandwidth specification for adaptive optics systems. J. Opt. Soc. Am..

[17] Robert J (1976). Noll. Zernike polynomials and atmospheric turbulence. J. Opt. Soc. Am..

